# Impact of Brain Surface Boundary Conditions on Electrophysiology and Implications for Electrocorticography

**DOI:** 10.3389/fnins.2020.00763

**Published:** 2020-08-07

**Authors:** Nicholas Rogers, Martin Thunemann, Anna Devor, Vikash Gilja

**Affiliations:** ^1^Department of Physics, University of California, San Diego, La Jolla, CA, United States; ^2^Department of Radiology, University of California, San Diego, La Jolla, CA, United States; ^3^Department of Neurosciences, University of California, San Diego, La Jolla, CA, United States; ^4^Martinos Center for Biomedical Imaging, Harvard Medical School, Massachusetts General Hospital, Charlestown, MA, United States; ^5^Electrical and Computer Engineering, University of California, San Diego, La Jolla, CA, United States

**Keywords:** electrophysiology, electrocorticography (ECoG), biophysics, device fabrication, neuroscience method

## Abstract

Volume conduction of electrical potentials in the brain is highly influenced by the material properties and geometry of the tissue and recording devices implanted into the tissue. These effects are very large in EEG due to the volume conduction through the skull and scalp but are often neglected in intracranial electrophysiology. When considering penetrating electrodes deep in the brain, the assumption of an infinite and homogenous medium can be used when the sources are far enough from the brain surface and the electrodes to minimize the boundary effect. When the electrodes are recording from the brain's surface the effect of the boundary cannot be neglected, and the large surface area and commonly used insulating materials in surface electrode arrays may further increase the effect by altering the nature of the boundary in the immediate vicinity of the electrodes. This gives the experimenter some control over the spatial profiles of the potentials by appropriate design of the electrode arrays. We construct a simple three-layer model to describe the effect of material properties and geometry above the brain surface on the electric potentials and conduct empirical experiments to validate this model. A laminar electrode array is used to measure the effect of insulating and relatively conducting layers above the cortical surface by recording evoked potentials alternating between a dried surface and saline covering layer, respectively. Empirically, we find that an insulating boundary amplifies the potentials relative to conductive saline by about a factor of 4, and that the effect is not constrained to potentials that originate near the surface. The model is applied to predict the influence of array design and implantation procedure on the recording amplitude and spatial selectivity of the surface electrode arrays.

## Introduction

Electrical activity of the brain is measured with a variety of methods, such as electroencephalography (EEG), electrocorticography (ECoG), and penetrating electrodes which have characteristics determined largely by the relative location of the electrodes to the various tissues of the head. Electrodes inserted into the brain can record the activity of individual neurons, while the spatial resolution of EEG is severely reduced by the volume conduction of the potentials through the cerebrospinal fluid (CSF), skull, and scalp. Correspondingly, it is an often-used approximation in intracortical electrophysiology to ignore tissue boundaries and to assume the medium is of infinite extent and homogeneous (Mitzdorf, 1985; Tenke and Kayser, 2012). However, when modeling EEG, the CSF layer, skull, and scalp must be included and the geometry of the tissue and electrodes has a large effect on the recordings or models (Tenke and Kayser, 2012; Rice et al., 2013; Vorwerk et al., 2014).

Although not impacted by superficial tissue layers of the head, intracranial electrophysiology is still subject to the effects of multiple tissue boundaries and properties. This has motivated studies on the effect of electrical potentials caused by tissue properties (Pettersen et al., 2006; Einevoll et al., 2007; Goto et al., 2010; Slutzky et al., 2010; Bleichner et al., 2011; Rice et al., 2013; Brodnick et al., 2019), the presence of the electrode and its effect on the surrounding tissue (Ollikainen et al., 2000; Blanche et al., 2005; Moffitt and McIntyre, 2005), or a combination of both effects (Zhang et al., 2006; von Ellenrieder et al., 2012; Ness et al., 2015; Hill et al., 2018). The scale of the effects described in previous work ranges from changes local to the electrode that alter the amplitude of single action potentials to whole-head EEG models altered by the presence of an insulating, subdurally implanted ECoG grid. Often the geometry of the boundaries is complex enough to entail use of finite-element methods (FEM) to obtain solutions.

The modality of electrophysiology perhaps most able to benefit from its own effect on the brain's potentials is electrocorticography (ECoG). The electrode-brain tissue boundary condition is determined by the material and geometry of the ECoG electrodes. Therefore, material and geometry changes can be used to modify the recording properties. The effect of the design and placement of ECoG electrodes on recorded action potentials was characterized in Hill et al. (2018). This general effect can be seen from predicted changes in EEG in Zhang et al. (2006) and ECoG (Ness et al., 2015) as well other ways in which the boundary is altered (Pettersen et al., 2006; Einevoll et al., 2007). In contrast to EEG, which is distant from the source of the potentials, and intracortical electrodes, which do not create a large boundary, ECoG has a large material footprint and may also be implanted very close to its target sources. We therefore expect the effect caused by the presence of the electrodes will be the largest in ECoG—especially in the case of shallow sources such as unit activity measured from the cortical surface.

The aim of this work is to quantify this effect both experimentally and theoretically. We tested the effect experimentally by implanting a laminar electrode array into the whisker barrel cortex of anesthetized mice while altering the brain surface boundary. This experimental setup allows convenient control of the boundary condition by use of a saline bath and it also measures the depth-dependence of the effect of the boundary conditions. The effect of boundary condition has been previously predicted (Nicholson and Freeman, 1975; Pettersen et al., 2006) and described (Einevoll et al., 2007). To quantify how the change at the brain surface impacts the potentials the averaged evoked response to whisker stimulation in the somatosensory cortex of mice was compared between conditions where the brain surface was dry (insulating) and when it was covered in artificial CSF (ACSF) which is roughly five times more conductive than brain tissue.

For the theoretical model we propose a planar model with three layers to allow us to include an intervening layer between the electrodes and the brain tissue. This model is applied to predict the effects of various intracranial electrode and experimental designs. The region of interest is small enough when using relatively shallow laminar electrodes or micro-ECoG arrays to allow us to create an analytically tractable model by assuming that the curvature of the brain surface can be neglected and that the lateral extent of the exposed cortex is large enough to avoid lateral edge effects. This model is applied to calculate the impact of the boundary condition on both laminar and ECoG electrode arrays.

## Methods

### Experimental Procedure and Analysis

#### Experimental Procedure

All animal work procedures were in accordance with a protocol approved by the Institutional Animal Care and Use Committees of UC San Diego (protocol S07360).

Adult mice were anesthetized with isoflurane and placed on a heating pad. A femoral artery was catheterized, and a tracheotomy was performed. An incision was made in the scalp to expose the skull. A ball electrode was inserted behind the skull under the scalp to be used as the reference electrode. The skull was fixed to the experimental frame with dental acrylic, and the acrylic was further used to build a well around and extending above the exposed skull. A craniotomy and durotomy were made above right whisker barrel cortex, roughly 3 mm in diameter. The well was filled with artificial CSF (ACSF) to prevent the exposed cortex from drying. The mice were put on artificial respiration prior to administration of pancuronium while blood pressure and CO_2_ were monitored. Anesthesia was switched to alpha-chloralose prior to stimulation, and the electrode array was inserted with its location determined either by single channel microelectrode (FHC, Inc., ME, USA) recording of evoked responses to whisker stimulation or based on stereotactic coordinates estimated from previous recordings.

**Figure 1:**
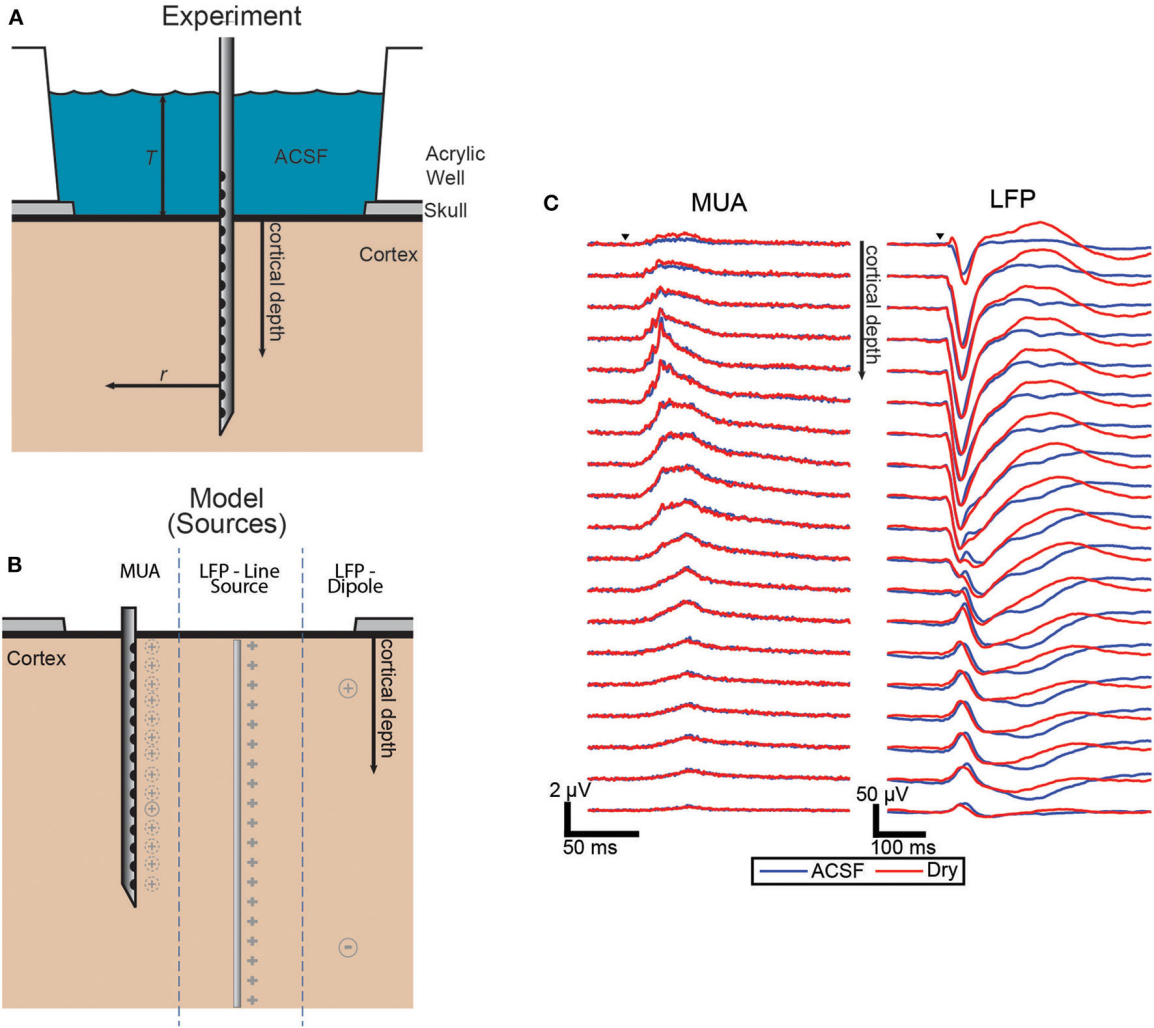
Experimental and model source schematic and evoked responses. **(A)** Schematic of the experiment with the laminar electrode inserted perpendicular to the surface and with the well either filled with ACSF or dried to vary the boundary condition. **(B)** Diagram of the three source configurations shown side-by-side with the locations shown in gray for each. The dashed lines around MUA indicate that each electrode has a different effective source location. **(C)** Grand averages (*n* = 43, each condition) of the evoked responses of both MUA and LFP signals. Trials covered with ACSF (blue) and dry (red).

The electrodes were laminar arrays with 22 contacts spaced 100 μm apart. The electrodes were inserted perpendicular to the cortical surface in or near whisker barrel cortex (Figure 1A).

Potentials were recorded using an Intan RHD2000 series amplifier and acquisition board (Intan Technologies, CA, USA) connected to the electrode array using a custom-built connector. The potentials were sampled at 20 kHz with the default hardware filters of the RHD2000: 0.1 Hz lower limit, 7.50 kHz upper limit and a DSP low cutoff of 1.0 Hz. The stimulus triggers were recorded simultaneously on one of the Intan system's ADC channels.

Single or multiple whiskers were stimulated using a wire loop glued to a piezoelectric actuator by placing the loop around the intended whiskers and deflecting the piezoelectric crystal with a 4 A sinusoidal pulse.

A set of trials consisted of 30–80 repetitions of the stimulus evenly spaced at 2 s intervals. Sets were paired by condition; first with the well above the exposure filled with ACSF, then repeated after the exposure was dried by wicking away the ACSF with a Kimwipe (Kimberly-Clark, TX, USA). The precise depth of the ACSF was not controlled between or within experiments, but the well would be filled several millimeters above the cortical surface immediately after each “dry” set. The model suggests that the effect of increasing the depth of the saline layer is negligible once the depth is greater than around 1 mm (Figure S1).

#### Signal Processing and Trial Selection

Multi-unit activity (MUA) was calculated by applying a high pass filter to the raw signal at 350 Hz and computing the amplitude of the Hilbert transform of the signal. The local field potential (LFP) was obtained using the raw signal downsampled to 4 kHz and only further filtered using a notch filter at 60 Hz for line noise removal. All signal processing and modeling was performed in MATLAB (Mathworks, MA, USA).

The most superficial electrode was determined from the data by visual inspection of the correlation matrix across electrodes of each “ACSF” trial. The first channel that was not nearly perfectly correlated (Pearson correlation coefficient slightly <1.0) with all channels located above it was determined to be the first, most shallow electrode in contact with the brain (see Figure S2).

Trial averages were computed for each set rather than averaged across sets with the same stimulus so that the comparison between the two boundary conditions was always made between an “ACSF” set and the successive “dry” set.

For each set a baseline MUA level was calculated by averaging the MUA signal from 0.09 to 0.01 s prior to the stimulus and subtracting this mean baseline level from the whole time series.

MUA signal is non-negative and the MUA responses are monophasic lending to a straightforward comparison of the magnitude of the average stimulus-evoked response. The evoked LFP has multiple positive and negative peaks and simply integrating the response results in temporal cancellation altering the measured ratio. Instead the RMS value over the window is used to estimate the overall magnitude of the LFP response to account for the relative amplitude of various oscillatory peaks. It is still an imperfect measure due to spatial cancellation that may result from the configuration of current sources.

The amplitude of a response was characterized by integrating the power of the signal over the duration of the response. For MUA this was calculated by taking the mean of the MUA signal after baseline removal from 0.01 to 0.09 s after stimulus onset. For LFP the response amplitude was determined by taking the RMS value of the signal between 0.01 and 0.4 s after stimulus onset.

Many sets of trials were recorded in which the whisker that was stimulated did not evoke an average response at the electrodes large enough to make a comparison between the two conditions. Sets were removed from further analysis if the averaged MUA response, after baseline subtraction, did not exceed 0.4 μV (above baseline) on any channel. After exclusion *n* = 43 pairs of ACSF/dry sets of trials were included in the follow analysis.

The comparison between the two conditions was computed as the ratio of the amplitude of the subsequent trials on a per trial, per electrode basis, and the effect as a function of depth was quantified by taking the median across trials for each contact depth. Significance of the median ratio being greater than or less than one was assessed by using two one-sided sign tests (above 1 and below 1) for the distribution of ratios on each contact.

The uncertainty in the depth of the first contact and the true locations of the current sources of the potentials prevents a direct quantitative comparison of the predicted and measured ratios.

### Biophysical Models

#### Three-Layer Model

We propose a planar three-layer model as an approximation of the geometry of intracranial electrophysiology near the brain surface. For sufficiently small electrode arrays we approximate the brain surface as flat and having no lower boundary as a lower half plane with homogenous isotropic conductivity 0.4 S/m (Goto et al., 2010). The brain is modeled as being covered by a uniformly thick layer of another material which is bounded from above by a completely insulating layer. In an acute experiment this layer represents the CSF [1.79 S/m (Latikka and Eskola, 2019)] layer above the brain which is open to air, and for a chronic experiment this approximates an arbitrarily thick layer of fluid or tissue covered by the insulating electrode array or approximating the skull which is relatively insulating [between one and two orders of magnitude less conductive than brain tissue (Vorwerk et al., 2014)] (Figure 1).

The sources of electric potentials in the brain are transmembrane currents (Plonsey, 1964). A small, localized transmembrane current, *I*, generates a potential throughout the volume of tissue with conductivity σ that has the familiar form a point charge in electrostatics,

$$\begin{array}{l} V=\frac{I}{4\pi \sigma r} \end{array}$$

where *r* is the distance from the source current and the conductivity replaces the permittivity.

The effect of the boundaries can be described by the modified Green's function for the three-layer model (see Appendix for derivation) which includes the usual source term as well as image sources whose magnitude and location are determined by the material properties and geometry.

$$\begin{array}{l} V_{1}\left(r,z,\phi \right)=\frac{1}{4\pi \sigma_{1}}[\frac{1}{\sqrt{r^{2}+{\left(z+D\right)}^{2}}}+\frac{\alpha }{\sqrt{r^{2}+{\left(Z-D\right)}^{2}}}\\ \;+\sum_{n=0}^{\infty }{\left(-\alpha \right)}^{n}\frac{1-\alpha^{2}}{\sqrt{r^{2}+{\left(z-D-2\left(n+1\right)T\right)}^{2}}}] \end{array}$$

$$\begin{array}{l} V_{2}\left(r,z,\phi \right)=\frac{1}{4\pi \sigma_{1}}\sum_{n=0}^{\infty }{\left(-\alpha \right)}^{n}\left(1+\alpha \right)[\frac{1}{\sqrt{r^{2}+{\left(z+D+2nT\right)}^{2}}}\\ \;+\frac{1}{\sqrt{r^{2}+{\left(z-D-2\left(n+1\right)T\right)}^{2}}}] \end{array}$$

where *D* is the depth of the source, *T* is the thickness of the intervening layer, σ_1_ is the conductivity of the brain, σ_2_ is the conductivity of the intervening layer, and α is the quantity

$$\begin{array}{l} \alpha =\frac{\sigma_{1}-\sigma_{2}}{\sigma_{1}+\sigma_{2}} \end{array}$$

The parameters can be altered to match a variety of conditions such as arbitrarily thick layers of either more or less conducting layers above the brain. The simpler one- or two-layer models can be modeled with no intervening layer as *T* approaches 0, no boundary condition as *T* approaches infinity. The two experimental conditions we tested are both modeled as having ACSF with the same conductivity as CSF (1.79 S/m) as the covering layer, but with the dry condition having a depth, *T*, of 0.001 mm and the ACSF condition having a depth of 5 mm.

#### Source Placement and ECoG Models

MUA recording was modeled by placing a source 50 μm laterally displaced from the electrode location (Blanche et al., 2005; Moffitt and McIntyre, 2005; Xing et al., 2009). This was accomplished by “moving” the sources for each electrode such that the relative location between the source and electrode is always the same (see Figure 1C). Sources are modeled as uniform 30 μm spherical current sources or sinks. This was done to keep the potential from diverging near the source, but to retain the potential of a point source outside of the 30 μm sphere.

A simple model is used to account for unknown and distributed sources of the LFP. The location of LFP-generating sources is not as easy to generalize as MUA and will always depend on the neuroanatomy and type of activity. A general model of the sources of LFP is a uniform vertical line charge, parallel to the electrodes and offset by 200 μm. This representation weights all cortical layers as contributing current sources to the potential and creates a relatively uniform potential across cortical depth. The LFP is approximated as being generated by a series of sources in a vertical line (perpendicular to the brain surface). This was approximated by 50 sources along the *z* axis spanning between 0.05 and 3 mm deep and 200 μm laterally displaced from the shank of the electrode. Due to the simplicity of the source geometry relative to real LFP, noise is added before taking the ratio which crudely includes the “noise” which would be due to all other sources not included in the modeled source.

A more realistic model is a current dipole with a sink and source pair. Their locations were chosen using an approximation of the actual sources based on current source density (CSD) analysis applied to the LFP (Figure S3). The actual current sources and sinks are expected to be spatially distributed and time-varying, but we chose to model only the largest sources and sinks visible as the only source/sink pair which were at depths 0.25 and 1.4 mm (Figure 1C). The pair was modeled as being laterally displaced 0.4 mm from the shank of the electrodes to represent the average effective distance to the various responsive whisker barrels as the whisker being stimulated was varied. As with the line source, additive noise is added prior to calculating the ratio between conditions.

The sensitivity of the electrodes was chosen as the metric which summarizes the effect of the boundaries and source locations and configurations (von Ellenrieder et al., 2012). It is a measure of the amplitude of the potential measured at fixed electrode location as a function of the position of a unit source, and accordingly it is measured in units V/A. The construction of sensitivity profiles is common in electrophysiology as the first step of many source localization algorithms that is carried out by modeling the magnitude of potentials induced at an electrode from locations of interest within the brain (Jonmohamadi et al., 2014). In our simple geometry this map is provided by the Green's functions for this boundary value problem. The sensitivity calculated this way is not true sensitivity of the electrodes which would include electrode effects such as electrode-tissue interface and the size and shape of that interface, and it is important to emphasize that this model represents volume conduction effects.

We chose to summarize two aspects of the sensitivity profiles that are important considerations for design and interpretation of the potentials. One is an estimate of the signal-to-noise ratio (SNR): with an estimate of the noise level present in the recordings and the size of the current source of interest, the sensitivity can be used to identify locations from which the response can be reliably measured. The second is the spatial specificity of the profile. Narrow sensitivity profiles may be desirable if we are interested in identifying the locations from which the potential originates, or broad profiles if detection is of more interest than localization.

To show the effect of altering the brain surface boundary conditions on the SNR we model a typical source for illustrative purposes. Based on our recordings and previous current density estimates (Higley and Contreras, 2007; Szymanski et al., 2011; Riera et al., 2012; Kajikawa and Schroeder, 2014) the source is assumed to be a current density of 40 μA/mm^3^ over a region of volume 0.0062 mm^3^ (volume of a 0.2 mm cube) which results in a source strength of 0.25 μA. We model the amplitude required to clearly detect this response strongly at an electrode as 100 μV. This defines a threshold sensitivity of about 400 V/A from which we can identify the cortical locations with respect to an electrode that would be expected to produce a clear response given these criteria.

It has been shown that an intervening saline layer can broaden laterally the sensitivity profile of surface electrodes (Ness et al., 2015; Hill et al., 2018). In a horizontal plane defined by a fixed depth the sensitivity has circular symmetry with a single peak directly under the electrode. To characterize this effect and the sensitivity profile we compute the half width at half maximum (HWHM) of the sensitivity as a function of source depth.

## Results

### Experimental Results

The effect of altering the boundary condition at the surface of the brain was measured using the ratio of the amplitude of the evoked responses in the ACSF-filled condition and the dry condition. To quantify the magnitude of the response for each condition pair, the trial averages were integrated over the duration of the evoked response. Figure 1B shows the grand average of the responses across all sets of trials for both MUA and LFP which both show the most difference between conditions near the surface with LFP differences extended to all depths.

**Figure 2:**
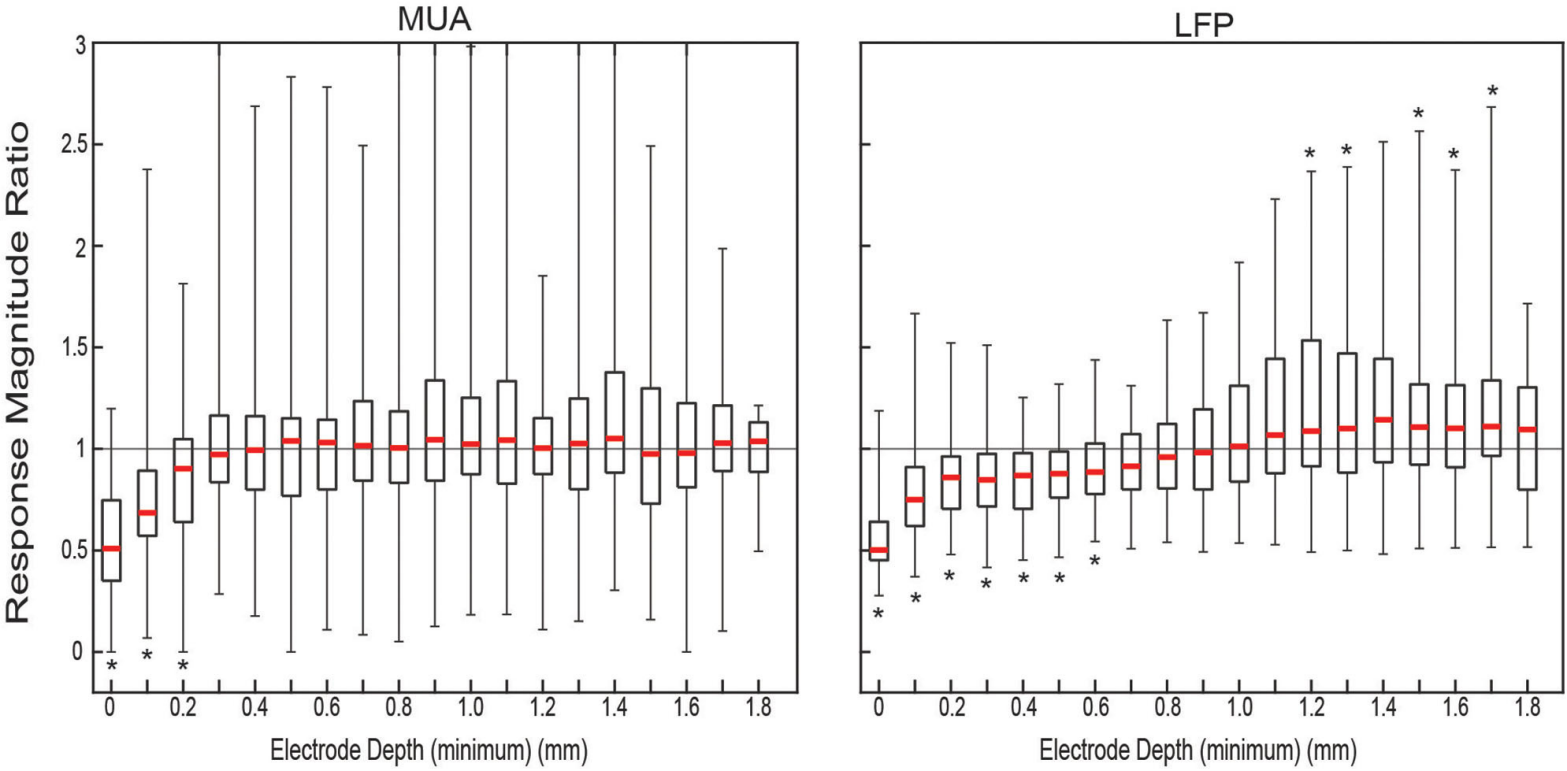
Measured ratio of evoked responses between conditions. Distributions of response magnitude ratio of all condition-pair sets of trials (*n* = 43). For each approximate electrode depth all the pairs (gray) and the quartiles (black) and median (red) of the distribution were plotted. Location of * denotes the median is significantly (*p* < 0.05) above (top) or below (bottom) 1.0.

The ratio of the responses was calculated per matched set of trials for each electrode depth. The distribution of ratios of the response magnitudes for both MUA and LFP, shown in Figure 2, showed clear attenuation for the ACSF sets relative to dry ones at the surface The median ratio of the MUA amplitude was significantly <1 (one-sided sign test *p* < 0.05) for the first three most shallow contacts. The ratio of the LFP amplitude showed significant relative amplification of the dry condition for the first seven contacts and attenuation at contacts 13 through 18—with the exception of contact 15 (not statistically significant).

### Laminar/Experiment Modeling

**Figure 3:**
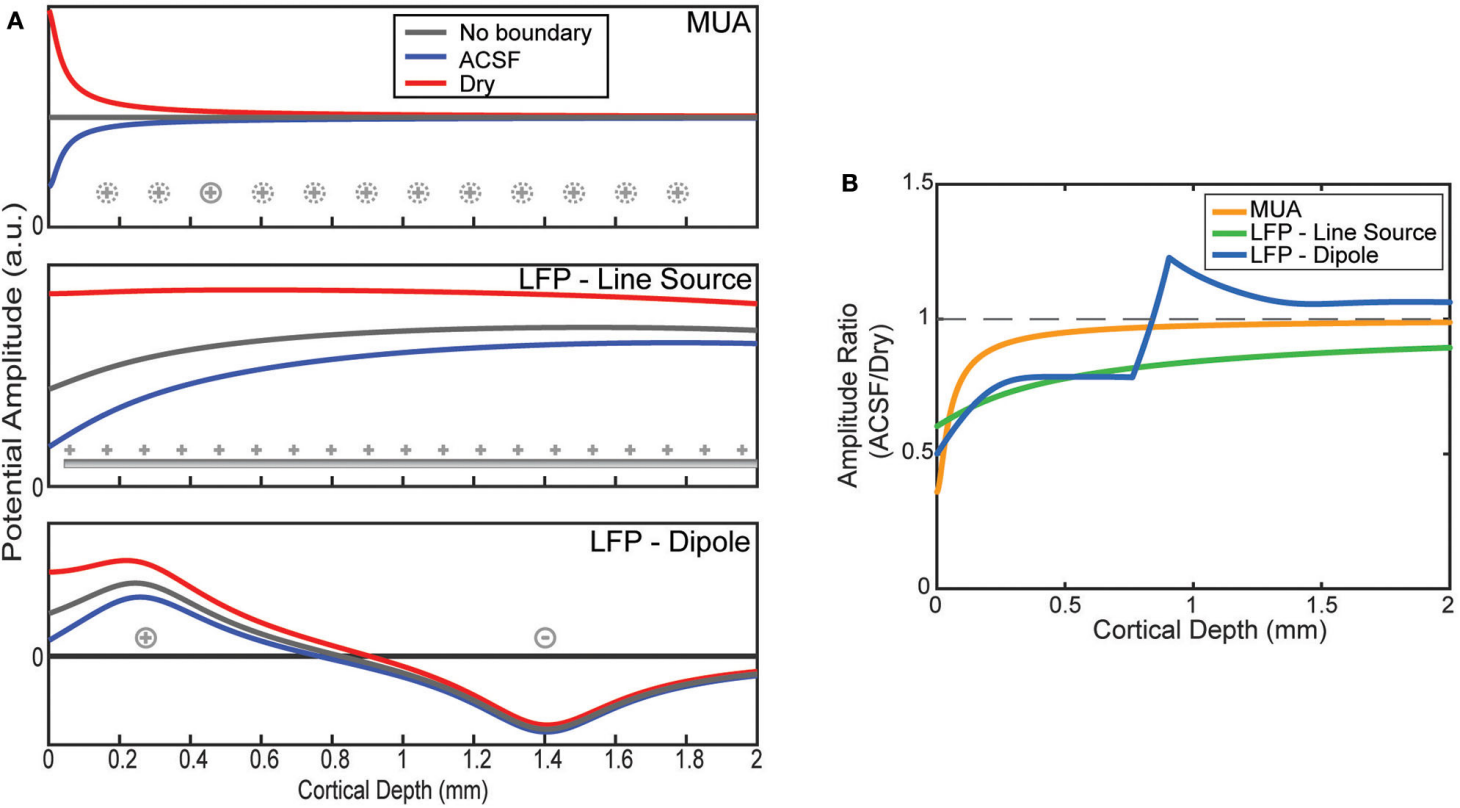
Model predictions of boundary on laminar electrode recordings. **(A)** Potentials as a function of depth are modeled by assuming a source configuration (as shown in Figure 1C). Depth profiles are plotted for an infinite medium and one bounded by ACSF or an insulator. **(B)** The ratios between the ACSF and insulating (dry) condition predicted by the model as a function of electrode depth.

The effect of the boundary condition on the laminar recordings depends on the locations of the sources being measured. The MUA model was constructed by placing the only source 50 μm from the position of each virtual electrode as shown in Figure 3A. Viewed as a ratio, the boundary effect on the MUA amplitude decays sharply from its largest effect near the surface attenuating the conducting condition by more than a factor of 2 but having no effect after about 1 mm of depth (Figure 3B).

LFP was modeled with two source configurations. First, the results for the uniform line source model show relatively flat potential profiles where the boundary is the only factor in altering the uniform profile (Figure 3A). The larger spread of sources causes a less pronounced effect at the boundary, but one that affects much deeper electrodes than MUA (Figure 3B). This agrees with the experimental results shown in (Figure 2) for the shallow electrodes but does not explain the effect measured at the deeper electrodes.

The more physically plausible model, using a dipole, is shown at the bottom of Figure 3A. The boundary effect on the amplitude is much larger near the surface, but that due to the change of depth at which the potential is zero there is a jump in the ratio between conditions (Figure 3B). Like the line source, the effect decays much more slowly with depth compared to MUA, but abruptly shifts to a small amplification near the depth of the deeper current.

**Figure 4:**
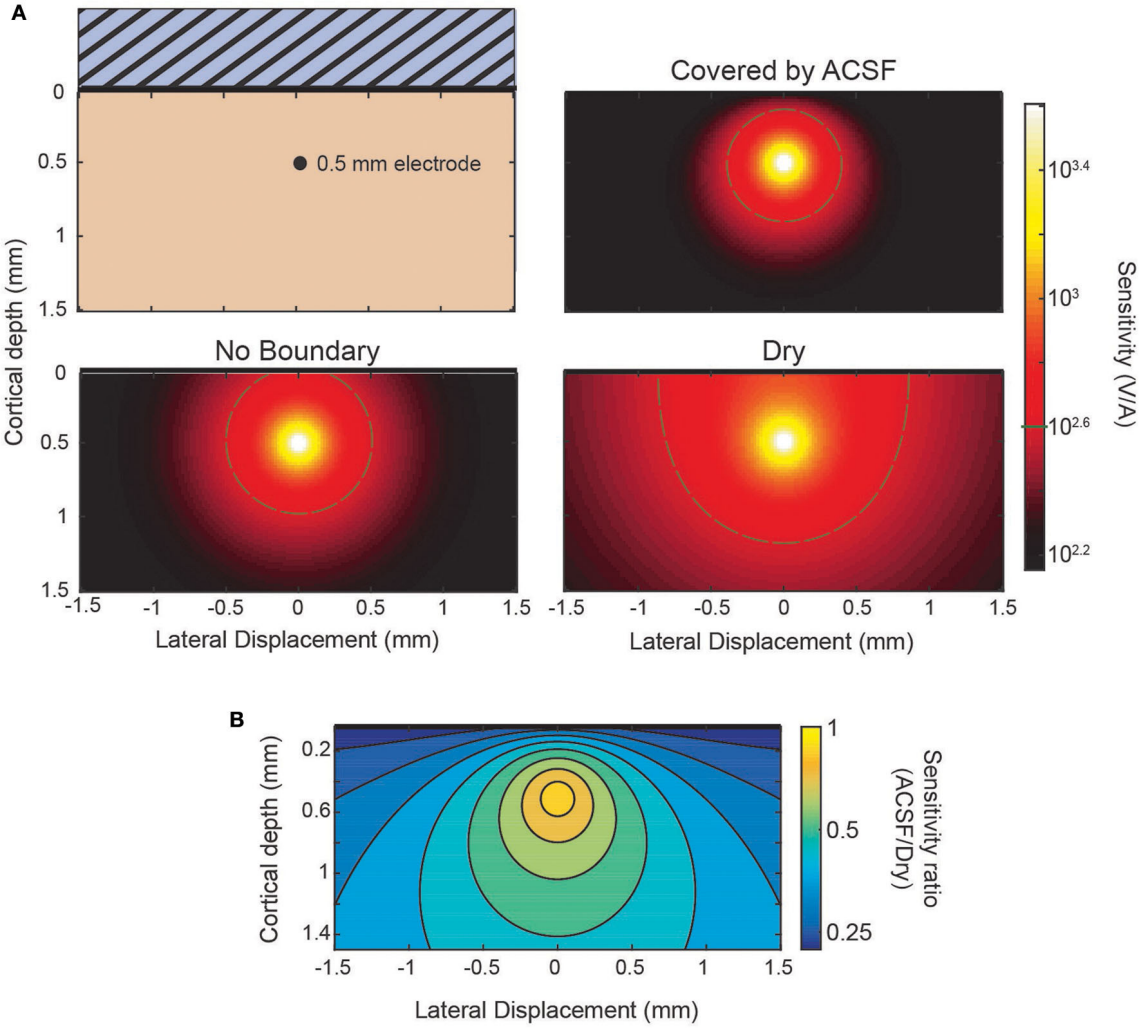
Sensitivity profiles of inserted electrode at 0.5 mm. **(A)** Lateral cross-section of the sensitivity of an electrode 0.5 mm deep. Comparison between the sensitivity without the presence of a boundary, with a conducting (ACSF) boundary, or an insulating (dry) boundary at the cortical surface. The 400 V/A threshold shown in gray. **(B)** The ratio of the ACSF and dry sensitivities from **(A)**.

The sensitivity profile of electrodes is a complementary analysis to the source-first application of the model. As an example of the use of the sensitivity profiles applied to the laminar electrodes, Figure 4 shows sensitivity profiles of the same three conditions from Figure 3: an infinite brain with no boundaries, a covering of ACSF, and a dry, insulating layer. It can be seen in the predicted laminar electrode sensitivities that the boundary condition more strongly affects sources located closer to the boundary while the more distant side is mostly unaffected. The attenuation of the shallow source causes less cancellation of the potential caused by the deeper sink. This can be seen in the ratio between conditions shown in Figure 4B where an electrode at the depicted depth of 0.5 mm would have little change in sensitivity to a source within 0.1 mm but would attenuate a shallower source. This change in the ratio between conditions shows the relative “screening” of the shallow sources created by the more conductive boundary that is also depicted in Figure 3.

### ECoG Modeling

Lastly, we apply the model to generate sensitivity maps for ECoG electrodes in different boundary conditions. We use the spatial map of the sensitivity of the electrodes as a method to compare the effects of electrode array designs and locations. These maps provide the input/output relationship between the location of a given source and the measured potential at the electrode, i.e., the gain of the electrode as a function of source location.

**Figure 5:**
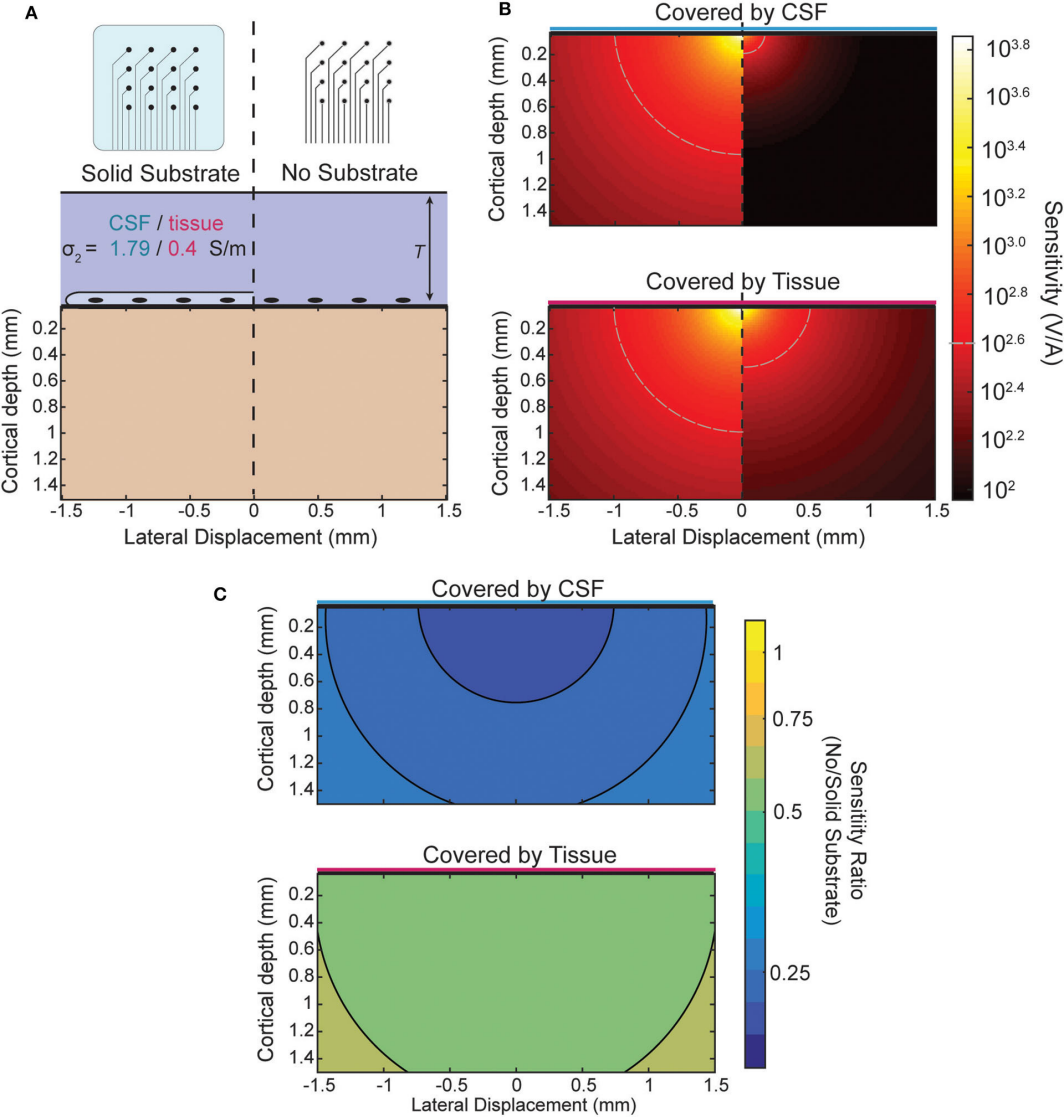
Comparison of sensitivity profiles of an electrode at the surface. **(A)** The design of the array and tissue in which it is implanted affect the sensitivity. Solid (insulating) arrays shown on left and minimal (no insulation) arrays on the right which can be covered by tissue or relatively conductive CSF. **(B)** Side-by-side comparison of effect of array type on sensitivity for CSF (top) and tissue (bottom). **(C)** Sensitivity ratio between array types for CSF or tissue above array.

When recording from an ECoG array the boundary condition is changed by the presence or lack of an insulating backing (illustrated in Figure 5A) and by the medium in which the array is implanted. The effect of the boundary on the sensitivity of an ECoG electrode is shown in Figure 5B for the four combinations possible between the two array types and media. The top of Figure 5B shows the sensitivity of both array types in, for example, an acute experiment where the array is submerged in CSF. The plot below shows the sensitivity when, as in a chronic implantation, the array is covered by tissue such as the dura. The ratio between the two types of array designs for each environment is shown in Figure 5C and is nearly uniform and modified by the properties of the tissue that covers the array. The level of attenuation is determined mostly by the conductivity ratio term α (see Appendix for derivation) which determines the strength and relative sign of the image sources. The approximate sensitivity ratio is the ratio of the total source (real plus image) between the two conditions

$$\begin{array}{l} \frac{Sens_{c}}{Sens_{i}\;}=\frac{1+\alpha_{c}}{1+\alpha_{i}} \end{array}$$

The values of α obtained using the conductivities of cortical tissue and CSF (see section Three-Layer Model) provide an estimate of the sensitivity ratio. For the insulating boundary α_*i*_ is +1 because the image source is of the same sign and magnitude. For tissue of similar conductivity to cortex α_*c*_ is roughly 0 because there is effectively no boundary and therefore no image source, and for CSF α_*c*_ is about −2/3. The resulting sensitivity ratios of ~0.5 and 0.33 can be seen in Figure 5C as the attenuation that results from the model and is nearly uniform.

**Figure 6:**
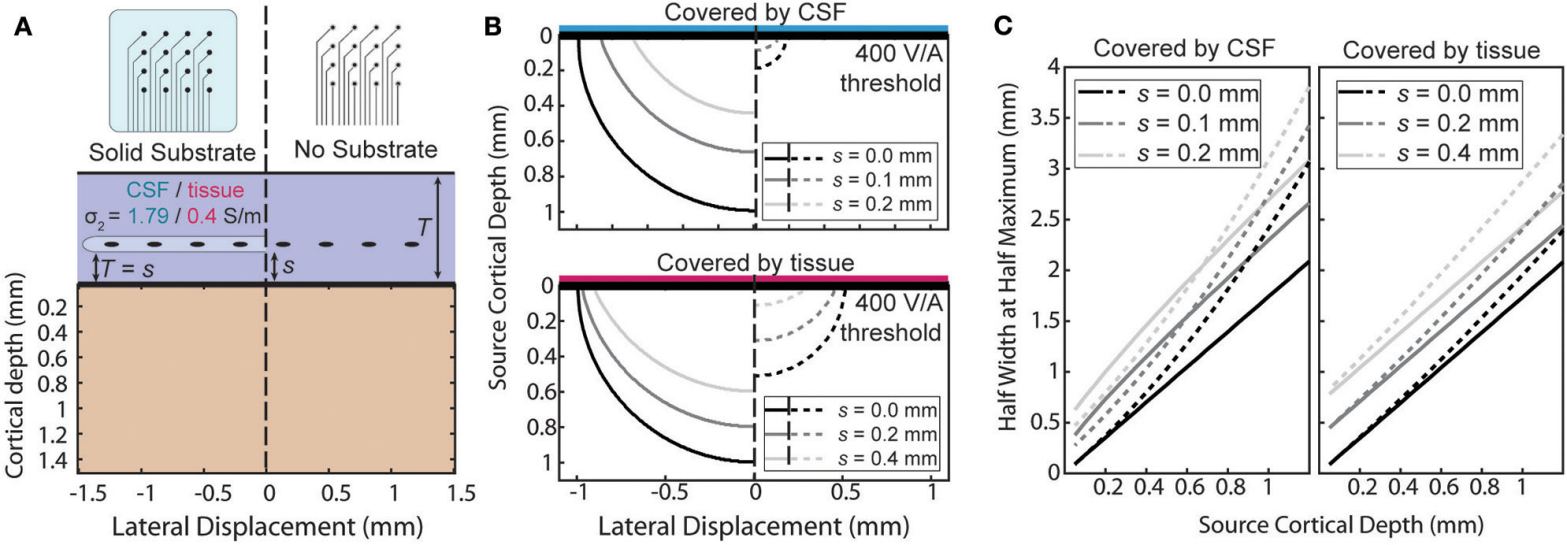
Effects of separation of the electrode above the surface. **(A)** The sensitivity with a separation, *s*, between the surface affects each condition differently. **(B)** Side-by-side comparison of the shape of the region with sensitivity >400 V/A for various separation distances. **(C)** Half width at half maximum of the sensitivity as a function of source depth at the same separations as **(B)**.

An ECoG array may not always lie directly on the pial surface. The array may be implanted above the dura or during chronic implantation scar tissue may grow under the array. When the array is not at the surface the ratio will no longer be uniform. The effect of the distance of separation, *s*, from the cortical surface and the boundary conditions is summarized in two ways in Figure 6. First, the size of the region above a threshold sensitivity is used to capture the effect on the magnitude of the sensitivity in Figure 6B. Second, the HWHM as a function of depth is used to describe the shape of the sensitivity profile in Figure 6C.

The amplification or attenuation caused by the boundary is described by the change in size or shape of the region in which the sensitivity is above some threshold value. Figure 6B shows the size of the region responsive at the 400 V/A threshold to a plausible source strength for the four combinations of array type and implantation medium and for three separation distances. The lack of an insulating layer at the ECoG array dramatically attenuates the signal for any thickness of intervening tissue, shrinking the “responsive” region. The effect is more pronounced when the material the array is implanted in is more conductive. The effect of increasing the separation of the array from the surface to also attenuate the signals, but the region shrinks less laterally than vertically indicating the effect is larger for deeper sources than lateral ones.

The horizontal broadening of the sensitivity profile is also an important characteristic of the potentials recorded by the electrode. This broadening, caused either by the depth of the sources or by changes in the materials or array, was summarized in Figure 6C using the half-width at half maximum (HWHM) of the sensitivity as the radial source displacement is varied. Volume conduction causes the profile to inevitably broaden as the sources become deeper, and separation of the contacts from the surface further increases the volume of tissue between the electrode and cortex. The Figure 6C shows that increasing the source depth always leads to an increase in HWHM as expected, and that increasing the separation between the array the surface increases the HWHM by a nearly constant amount across all source depths.

## Discussion

### Laminar Electrodes

The model and the experimental results suggest that the effect of CSF compared to an insulating layer above the cortical surface is limited to contacts near the surface. This agrees with the modeling of single units in Hill et al. (2018) which predicts smaller peak-to-peak amplitudes by about a factor of four when the electrode is small and has no insulating layer.

There are clear differences in the evoked LFP response to both conditions. In our LFP and in Einevoll et al. (2007) the effect is not constrained to the surface but is most apparent in the appearance of an early positive deflection near the surface. The broad influence of the boundary was realized in an early CSD study in which the experimental application of insulating mineral oil to the surface was accounted for in the authors' semi-infinite, two-layer model by simply multiplying all the potentials uniformly by a factor of two (Nicholson and Freeman, 1975).

When conductive media are above the surface the changes in LFP due to the boundary are more difficult to model or quantify. The current sources creating the potentials need to be mapped in order to predict the changes precisely. This could be accomplished from the recorded LFP, but accurate source localization in electrophysiology is itself the subject of study, and furthermore the effect of the boundary would itself need to be incorporated into the localization model. In the model this is accounted for either by use of a spatially “neutral” line source as an approximation of perhaps the average of many heterogeneous responses across trials, regions, or even species.

In the experimental results we see some amplification rather than attenuation of LFP for the more conductive boundary. The experimental results are best approximated by a model that used a plausible source configuration. This is explained by the greater attenuation of the source or sink closer to the surface of a dipole pair which acts to mask its contribution to the potential at a deeper electrode thereby reducing its cancellation of the potential of the deeper source/sink. The qualitative difference between the two models of LFP highlights the importance of source configuration. Sensitivity provides an electrode-centric rather than source-centric view of volume conduction, but the importance of the source configuration should not be neglected. This can be seen in EEG sensitivity models in which the assumed sources are dipoles rather than monopoles and this results in a different, but complementary, sensitivity profile despite describing the same phenomenon.

### ECoG Model

It is conventional wisdom that electrode sensitivities are largest near the contact and that minimizing the separation of surface electrodes from the surface improves the recordings, but our model predicts that the type and relative positioning of the boundary influenced by an ECoG array has effects that are much broader than just edge effects. The modeled effect is large and predicts that there is a strong advantage in embedding the electrodes in large insulating substrates as predicted by Hill et al. (2018) for spiking activity, but that this holds true for LFP recordings as well. The nearly spatially uniform attenuation is due to the electrodes always recording from the boundary where the edge effects apply. The factor of ~2 attenuation caused by the ACSF well at the top contact of the laminar array for both MUA and LFP agrees with the model assuming the LFP is largely caused by deeper sources.

It is often the case that it is undesirable or not possible for ECoG arrays to be implanted in contact with the pial surface, and our three-layer model allows for the effect of an intervening layer to be included. The conductivity and separation values as well as the use 400 V/A threshold and HWHM were chosen to demonstrate the main predictions for common scenarios, and the values can easily be modified to fit other experimental settings. The use of a threshold sensitivity has value in allowing a notion of signal-to-noise ratio (SNR) to be included in the sensitivity analysis. The sensitivity profile alone seems to suggest that every source will be recorded by an electrode, and it is only a matter of the amplitude of the potential. This may be true in principle, but in practice any constellation of sources of interest create potentials that exist in a background of other activity that is temporally and spatially interrelated and complex (Herreras, 2016) (neural or artifact) that can be broadly categorized as noise. With an understanding of the level of this noise and the magnitude of the current sources of the desired activity, the sensitivity threshold can be estimated to identify responsive regions in the brain.

The HWHM of the sensitivity also has a practical application for array design because it defines the electrode spacing at which a source directly under one electrode will cause a potential with half the amplitude at a nearest neighbor electrode within an array. With an application in mind, this provides a heuristic approach to layout ECoG electrodes to perhaps minimize signal redundancy or, conversely, to help ensure a desired signal is recorded strongly by multiple electrodes. When turned toward optimizing ECoG layouts based on spatial properties of the signal (Hermiz et al., 2018) the difficulty is disentangling coherence across space caused by volume conduction and caused by distinct sources which are themselves causing coherent signals. As in Slutzky et al. (2010), volume conduction, as described by the sensitivity of the electrodes is the unavoidable baseline level at which potentials at the surface will be correlated which will then be modified by the coherence between the set of sources and their distributed locations.

**Figure 7:**
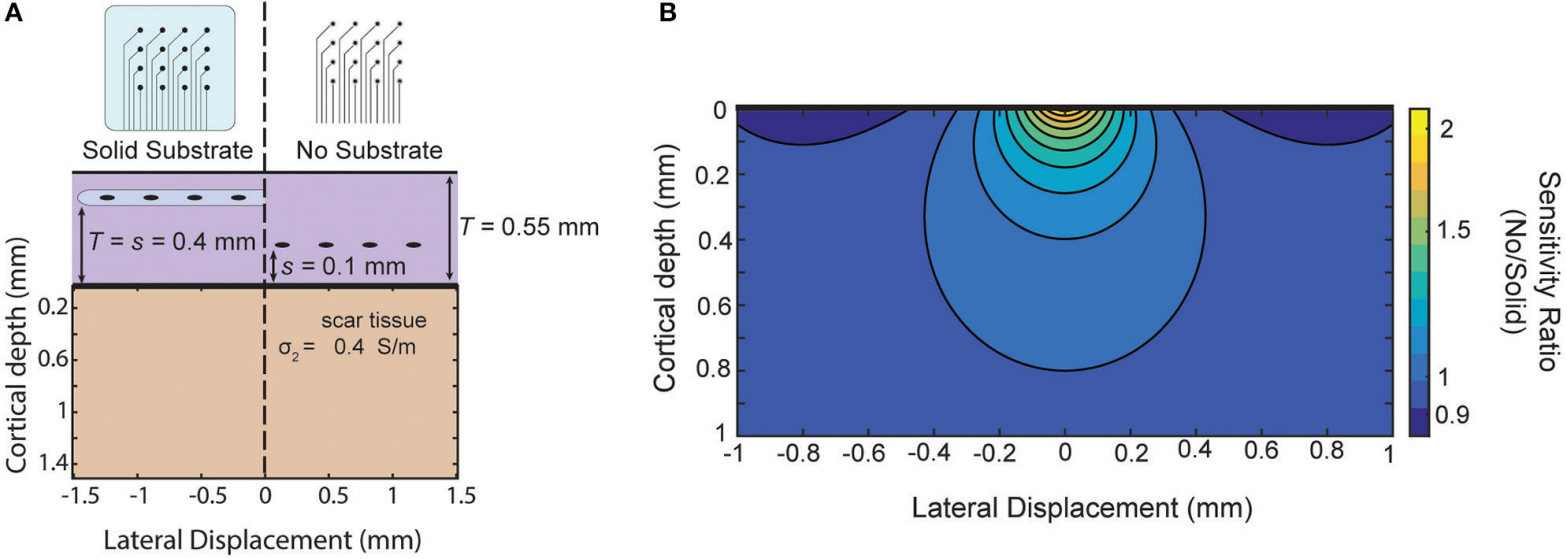
Comparison of sensitivity of two array designs for chronic implantation. Comparison of two electrode array designs which impact scar tissue formation as shown in Schendel et al. (2014). Using scar tissue thickness reported by the authors the model predicts the sensitivity ratio between the two arrays after tissue growth if we use our simplified model that the “mesh” electrode does not cause any insulation effects.

As an example, in Schendel et al. (2014) ECoG arrays with different amounts of insulation are implanted chronically. Their results show the footprint of the array impacts the growth of scar tissue above and below the array. As an example of this approximate model in guiding future array designs, we use these measured thicknesses to model the relative sensitivity of our approximation of the two designs (Figure 7A). The solid substrate is assumed to extend far past the edge of the array and the “mesh” array is approximated as not creating any insulating effect. For these parameters, the model favors the use of the “mesh” array due to the larger sensitivity near the surface and only modest attenuation at larger distances as shown as a ratio in Figure 7B. The success of recording micro-ECoG through a thinned skull (Brodnick et al., 2019) also motivates extending the model to four layers to make predictions about the properties of thinned skull ECoG. Still, intuition gained from our model suggests the limitation of that method is not the very thin layer of bone, rather it is the much thicker layers of tissue and conductive CSF between the array and cortical surface [similar to the effect on EEG in Rice et al. (2013)].

In our model the near uniformity is also a consequence of the simplifying assumptions of infinite depth and lateral extent of the gray matter. Even in our simple model the scale of the effect as a function of depth is limited by the thickness of the middle layer of CSF/tissue. For sources much deeper than this thickness the boundary begins to appear increasingly like a simple insulating boundary, and the ratio approaches one. The thickness of the tissue/CSF layer is often much thicker than cortex, where the sources of interest are concentrated in micro-ECoG, but this does suggest that arrays lacking insulation may record LFPs that are largely unaffected by the lack of insulation if they are covered by a layer of material that is thinner than the depth of the sources of interest. More concretely, if the array is covered by less than a millimeter layer of saline in an open exposure or is implanted within a millimeter of the skull, the insulation begins to have less effect on cortical sources due to the nearby naturally insulating boundaries.

The approximations limit the application of our results to sources whose depth is much less than the extent of the insulation of the array, the size of the craniotomy, or the radius of curvature of the cortical surface. If limited to applications of micro-ECoG and measuring cortical activity, the latter approximations will generally be valid. We can apply scaling arguments to the results of Hill et al. (2018) to understand the effect of finite insulating are on deeper sources. Using FEM models to vary the size of the insulation area around the electrode, they predicted that for a 20 μm deep source it takes 30 μm of lateral insulation to maintain 95% of the fully insulated amplitude. Under the approximation that the array is covered by a very deep layer of CSF and in a very large craniotomy these results suggest that the size of the insulating layer has a small impact as long as the insulation extends in any direction more than 50% farther than the depth of the sources.

As ECoG arrays continue to be developed their design has been influenced largely by mechanical, physiological, and optical considerations. Electrode arrays affect the electrical conduction of the signals, and there is a complex interplay between all these factors in determining the qualities of the recordings. The impact of implanting the devices, which may be minor, of limited extent, and/or difficult to modify in other modalities, is significant in ECoG and should be a major consideration in the design of the arrays.

## Data Availability Statement

The datasets generated for this study are available on request to the corresponding author and available online at https://doi.org/10.6084/m9.figshare.11827821.v1.

## Ethics Statement

The animal study was reviewed and approved by Institutional Animal Care and Use Committees of UC San Diego (protocol S07360).

## Author Contributions

NR and MT performed experiments and collected data. NR created the models, developed the analysis, and wrote the manuscript. VG and AD oversaw modeling, data analysis, advised aspects of experiment design and data collection methods, and assisted with manuscript preparation. All authors contributed to the article and approved the submitted version.

## Conflict of Interest

VG holds shares in Neuralink, Corp. and Paradromics, Inc. and currently consults for Paradromics, Inc. The remaining authors declare that the research was conducted in the absence of any commercial or financial relationships that could be construed as a potential conflict of interest.

## Supplementary Material

Figure S1 Pairwise correlation of all electrodes. Correlation values calculated pairwise between all electrodes while the brain was covered in ACSF. The channels are numbered from top to bottom. The electrodes above the surface are shorted by the ACSF and show a notably higher correlation across all exposed electrodes. Values shown are for one example trial in which the most shallow electrode was determined to be the 4th electrode from the top.

Figure S2 Effect of finite saline depth. Model predictions of the ratio between MUA response for a dry surface compared to a finite layer of ACSF covering the brain of varying thickness. Shallower ACSF layers show less attenuation with the effect approaching the infinite depth case at around 1.0 mm.

Figure S3 Current source density of the response. **(A)** Grand average of all LFP responses (same as Figure 1B) plotted as a colormap with deeper contacts shown lower but voltage represented by color intensity. **(B)** Current source density (CSD) estimate obtained with the 2nd derivative of the LFP shown above with color now representing current sources and sinks.
