# Supplementary figures and images for: Impact of Brain Surface Boundary Conditions on Electrophysiology and Implications for Electrocorticography
Source: Front Neurosci. 2020 Aug 7;14:763. doi: 10.3389/fnins.2020.00763 (PMC7438758; doi:10.3389/fnins.2020.00763)

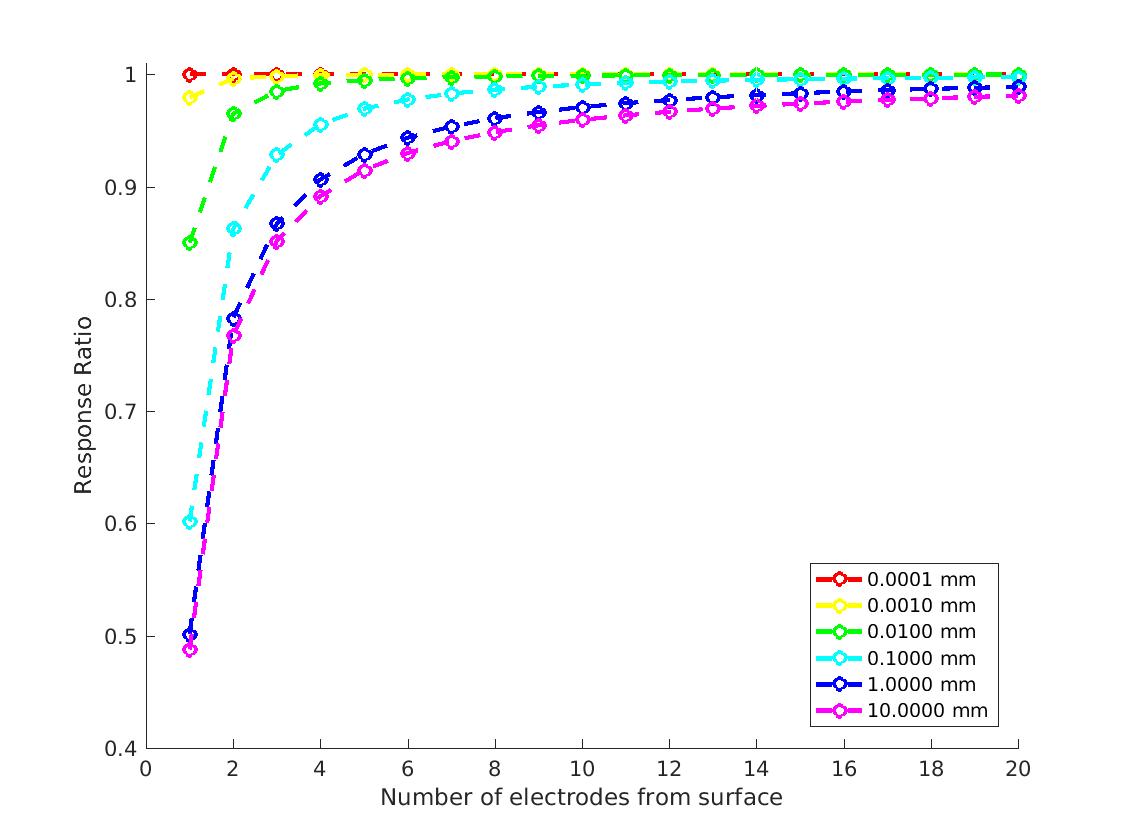

Supplement: Figure S1 — Pairwise correlation of all electrodes. Correlation values calculated pairwise between all electrodes while the brain was covered in ACSF. The channels are numbered from top to bottom. The electrodes above the surface are shorted by the ACSF and show a notably higher correlation across all exposed electrodes. Values shown are for one example trial in which the most shallow electrode was determined to be the 4th electrode from the top. [file Image_1.JPEG]

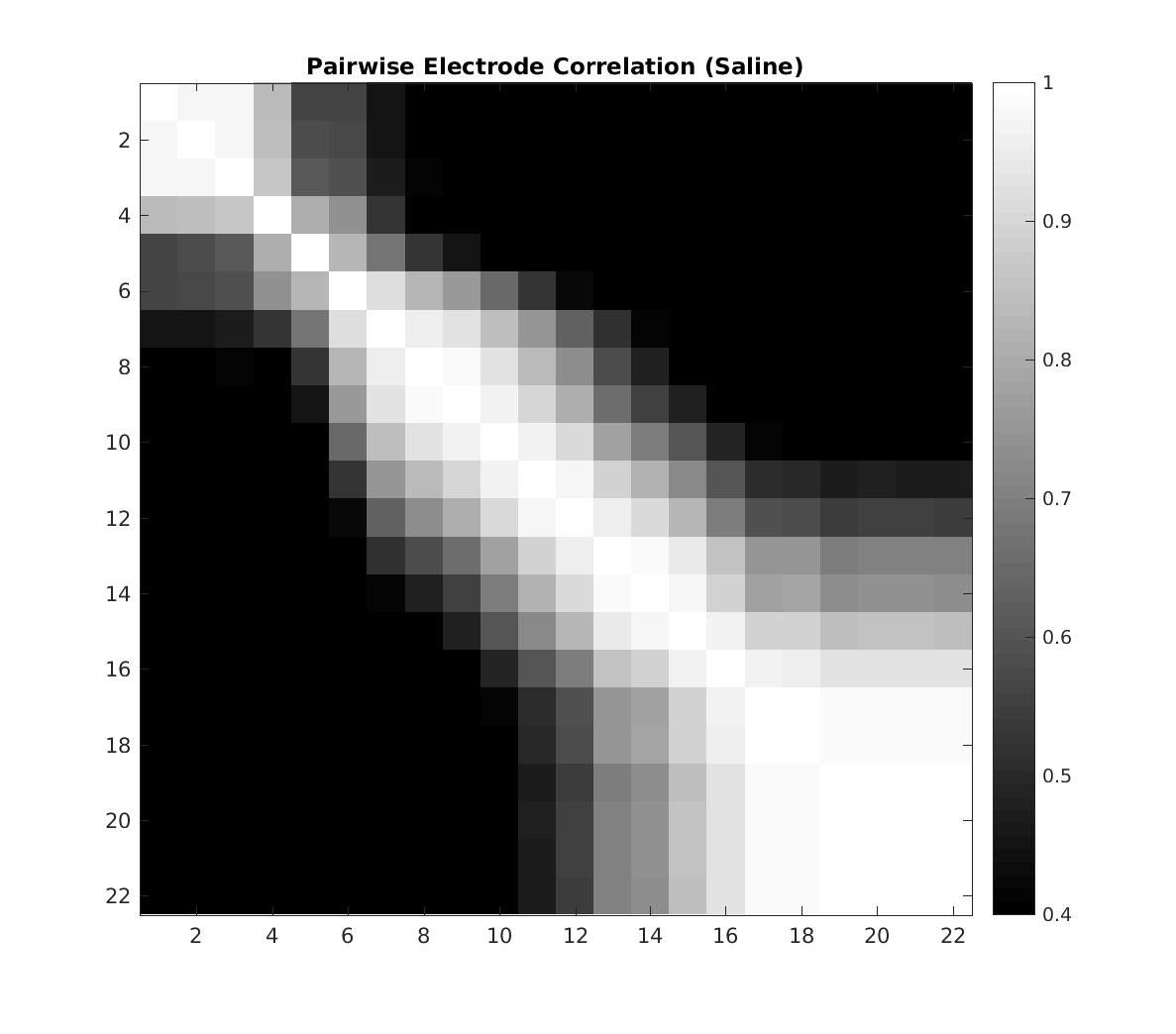

Supplement: Figure S2 — Effect of finite saline depth. Model predictions of the ratio between MUA response for a dry surface compared to a finite layer of ACSF covering the brain of varying thickness. Shallower ACSF layers show less attenuation with the effect approaching the infinite depth case at around 1.0 mm. [file Image_2.JPEG]

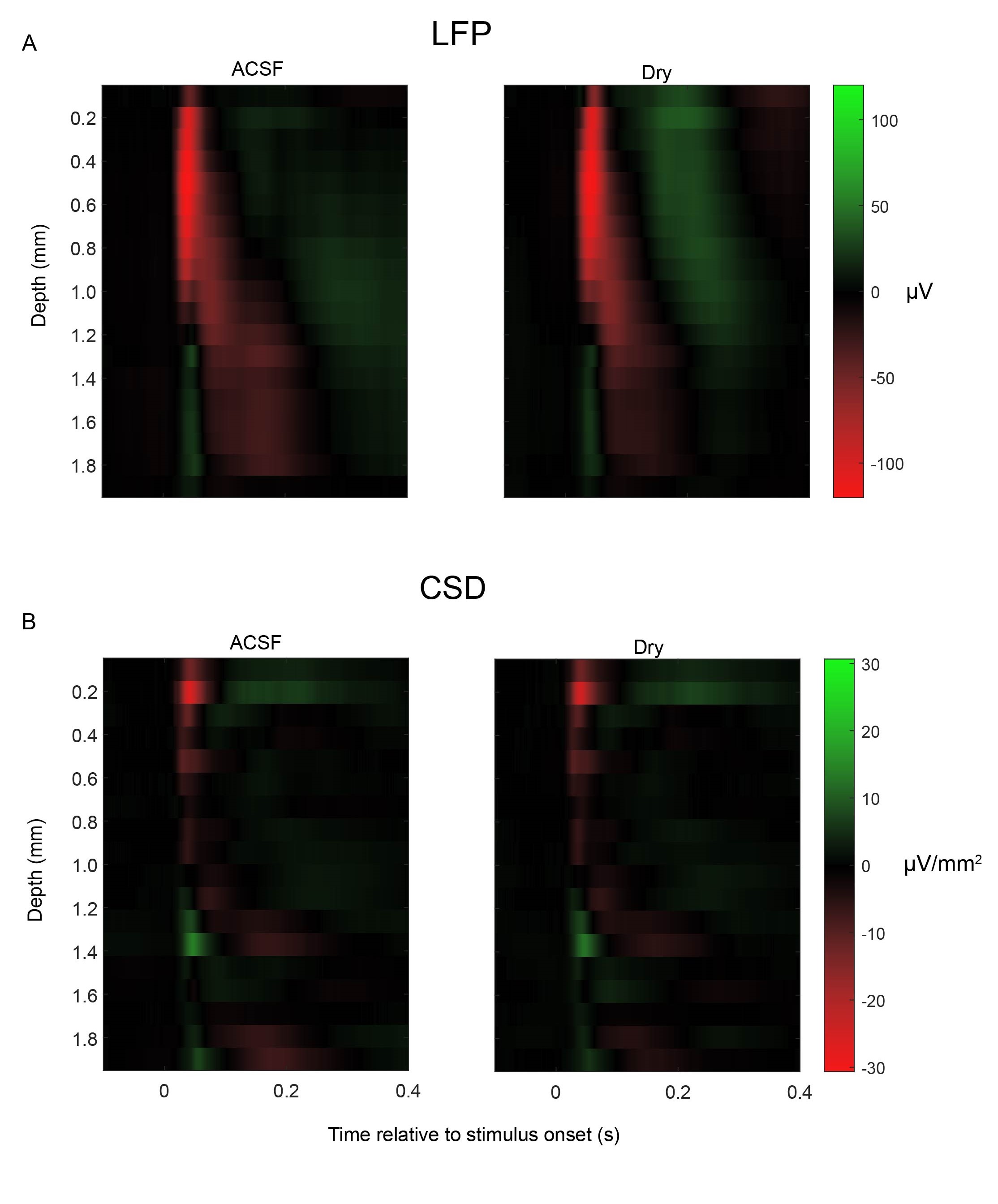

Supplement: Figure S3 — Current source density of the response. (A) Grand average of all LFP responses (same as Figure 1B) plotted as a colormap with deeper contacts shown lower but voltage represented by color intensity. (B) Current source density (CSD) estimate obtained with the 2nd derivative of the LFP shown above with color now representing current sources and sinks. [file Image_3.JPEG]
