# Supplementary material for: Impact of Brain Surface Boundary Conditions on Electrophysiology and Implications for Electrocorticography
Source: Front Neurosci. 2020 Aug 7;14:763. doi: 10.3389/fnins.2020.00763 (PMC7438758; doi:10.3389/fnins.2020.00763)
Supplement: Supplementary file 4 [file Data_Sheet_1.docx]

Appendix

To simplify the geometry involved we will treat the cortical surface as having no curvature, and that the cortex is uniform, isotropic, and extends infinitely far down. We set $z = 0$ at the cortical surface, and assume there is a conductive medium with uniform thickness above the cortical surface. We assume that above the 2nd medium is a perfect insulator which approximates air in recordings where the cortex is exposed.


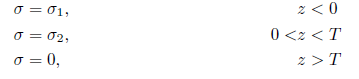


We wish to find the solution to a point source inside the cortex at a depth $D$. The potential in each region is a solution to


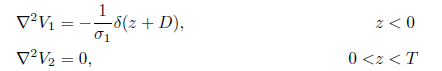


The horizontal extent of the medium is considered infinite, and at the potential should go to zero for for locations distant from the origin. The potential must have no discontinuities, and therefore must match at the boundaries. Charge conservation relates the normal derivatives of the potentials at the boundaries.


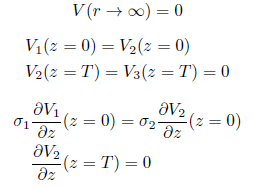


We choose to use cylindrical coordinates due to the radial symmetry of the source and boundaries. Using separation of variables, the solutions to Laplace's equation in cylindrical coordinates are given by linear combinations of the products of Bessel functions of $r$, and exponential or sinusoid functions of $z$. Symmetry of the source restricts the solutions to having no angular dependence, and significantly simplifies the general form of the solution by only allowing Bessel functions of order 0. In both media the boundary conditions require that the potential is finite everywhere and approaches 0 for large distances. Therefore the radial component of the complementary solutions can only be Bessel functions of the first kind with order 0, $J_0$. The particular solution for $V_1$ is the sum of the well-known solution to Laplace's equation for a delta function and the complementary solution.


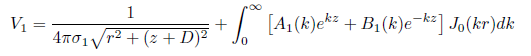


The general solution above will satisfy Laplace's equation everywhere except at the point source, where it will equal the delta function source. The boundary conditions can be satisfied by finding the appropriate $A_1(k)$, and $B_2$ must be zero for the solution to remain finite as $z \to - \infty$.

In the 2nd medium there are no sources, and the solutions are the homogeneous solutions, as above.


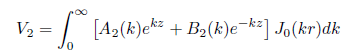


The condition at the top, $z=T$, that $V_2 = 0$, requires that $B_2(k) = e^{2kT}A_2(k)$. The boundary conditions at $z=0$ combined with the identities


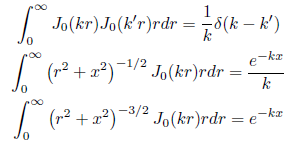


determine the potentials, and results in solutions for the coefficients as


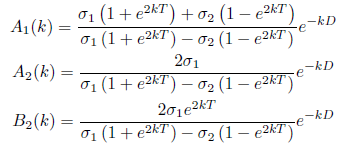


The integrals that make up the solutions can be evaluated using


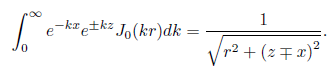


The coefficients can be written as sums using the series


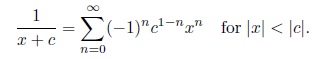


By breaking the the integrals into sums and evaluating them we obtain a much simpler form for the potentials. < Missing 4 pi sigma terms >


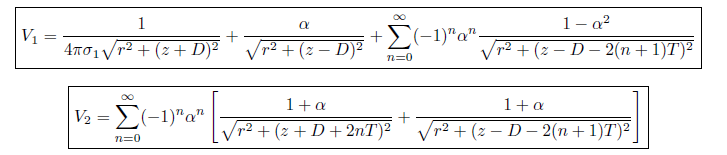


The constant $\alpha$ reflects the relative conductivity difference between the two media.


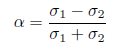


The form of the solution makes it apparent that the solutions are of the form of the method of images, but in with the extra layer requires an infinite number of image charges.
